# Factors affecting progression of non-Alzheimer dementia: a retrospective analysis with long-term follow-up

**DOI:** 10.3389/fneur.2023.1240093

**Published:** 2023-10-18

**Authors:** Reza Ghouri, Nevra Öksüz, Bahar Taşdelen, Aynur Özge

**Affiliations:** ^1^Department of Neurology, School of Medicine, Mersin University, Mersin, Türkiye; ^2^Department of Biostatistics, School of Medicine, Mersin University, Mersin, Türkiye

**Keywords:** non-Alzheimer’s dementias, temporal course, vascular dementia, frontotemporal lobar degeneration, Lewy body dementia, Parkinson’s disease dementia, hippocampal atrophy, disease progression

## Abstract

**Background:**

Non-Alzheimer’s dementias, including vascular dementia (VaD), frontotemporal dementia (FTD), Lewy body dementia (LBD), and Parkinson’s disease dementia (PDD), possess unique characteristics and prognostic factors that remain poorly understood. This study aims to investigate the temporal course of these subtypes and identify the impact of functional, neuropsychiatric, and comorbid medical conditions on prognosis. Additionally, the relationship between hippocampal atrophy, white matter intensities, and disease progression will be examined, along with the identification of key covariates influencing slow or fast progression in non-Alzheimer’s dementias.

**Methods:**

A total of 196 patients with non-Alzheimer’s dementias who underwent at least three comprehensive evaluations were included, with proportions of VaD, FTD, LBD, and PDD being 50, 19.39, 19.90, and 10.71%, respectively. Patient demographics, comorbidities, neuropsychiatric and neuroimaging parameters, and global evaluation were analyzed using appropriate statistical methods. The study followed patients for a mean duration of 62.57 ± 33.45 months (ranging from 11 to 198 months).

**Results:**

The results from three different visits for each non-AD dementia case demonstrated significant differences in various measures across visits, including functional capacity (BDLAS), cognition (MMSE), and other neuropsychological tests. Notably, certain genotypes and hippocampal atrophy grades were more prevalent in specific subtypes. The results indicate that Fazekas grading and hippocampal atrophy were significant predictors of disease progression, while epilepsy, extrapyramidal symptoms, thyroid dysfunction, coronary artery disease, diabetes mellitus, hypertension, stroke, hyperlipidemia, sleep disorders, smoking, and family history of dementia were not significant predictors. BDLAS and EDLAS scores at the first and second visits showed significant associations with disease progression, while scores at the third visit did not. Group-based trajectory analysis revealed that non-AD cases separated into two reliable subgroups with slow/fast prognosis, showing high reliability (Entropy = 0.790, 51.8 vs. 48.2%).

**Conclusion:**

This study provides valuable insights into the temporal course and prognostic factors of non-Alzheimer’s dementias. The findings underscore the importance of considering functional, neuropsychological, and comorbid medical conditions in understanding disease progression. The significant associations between hippocampal atrophy, white matter intensities, and prognosis highlight potential avenues for further research and therapeutic interventions.

## 1. Introduction

Dementia is a progressive decline in cognitive function that arises due to brain damage or disease, representing one of the most significant causes of elderly disability worldwide. It ranks as the third leading cause of death and imposes a substantial socio-economic burden across the globe. Dementia encompasses various types, including Alzheimer’s disease, vascular dementia, frontotemporal dementia, and Lewy body dementia, all of which necessitate a rigorous exclusion of secondary causes (1).

Vascular dementia (VaD) emerges as a dementia variant triggered by diminished or obstructed blood flow to the brain. This reduction in blood circulation can result in brain damage and cognitive decline, involving intricate processes. VaD stands as one of the prevalent dementia types after Alzheimer’s disease. The latest diagnostic criteria for VaD are outlined in the 2013 publication titled “Vascular Cognitive Impairment Harmonization standards” by the Vascular Dementia Harmonization Group. These criteria characterize VaD as a decline in cognitive function attributed to cerebrovascular diseases, including stroke, white matter disease, and microvascular disease. The criteria underscore the importance of considering the patient’s medical history, imaging studies, and laboratory tests to diagnose VaD (2, 3).

Frontotemporal dementia (FTD) constitutes a cluster of neurodegenerative disorders characterized by progressive deterioration in the frontal and temporal brain lobes. This degeneration gives rise to alterations in personality, behavior, and language capabilities. FTD, accounting for approximately 5–10% of dementia cases, is less prevalent compared to other forms. FTD is defined by a progressive decline in behavior or language skills, or both, distinguishing it from conditions like Alzheimer’s disease. The criteria also encompass neuropathological guidelines for diagnosing FTD, founded on distinct patterns of brain degeneration (4).

Lewy body dementia (LBD) denotes a neurodegenerative disorder marked by the presence of irregular protein aggregates referred to as Lewy bodies within the brain. LBD leads to gradual cognitive and motor function decline and frequently correlates with visual hallucinations and fluctuations in attention and alertness. Diagnostic criteria identify LBD as a dementia syndrome characterized by core traits, including fluctuating cognition and attention, recurrent visual hallucinations, and parkinsonism. The criteria also stress the importance of considering the patient’s medical history, imaging studies, and laboratory tests for an accurate LBD diagnosis (5).

Parkinson’s disease dementia (PDD) arises in individuals with Parkinson’s disease, a progressive nervous system disorder affecting movement. PDD is recognized by the coexistence of Parkinson’s symptoms like tremors, bradykinesia, stiffness, and motor impairment, alongside cognitive decline encompassing memory loss and cognitive challenges. Diagnostic criteria define PDD as a clinical syndrome occurring in established Parkinson’s disease cases, featuring progressive cognitive function decline, including memory, attention, and executive function, with or without accompanying motor function decline. The criteria emphasize the significance of comprehensive clinical evaluation and review of the patient’s medical history for accurate PDD diagnosis (6).

Dementia progression varies across distinct types. For example, Alzheimer’s disease typically advances gradually over several years, with symptoms worsening over time (2). On the contrary, VaD might have a swifter onset and progression, often triggered by a stroke or another vascular event (7). FTD, impacting the frontal and temporal brain lobes, generally advances more rapidly than Alzheimer’s disease, with symptoms deteriorating over months to years (4). LBD, characterized by symptoms resembling both Alzheimer’s and Parkinson’s diseases, exhibits variable progression (5). It is vital to recognize that progression varies individually and might hinge on factors such as age, genetics, and overall health.

The advancement of non-Alzheimer’s disease (non-AD) dementia is influenced by diverse factors, including neuropsychiatric, radiologic, and biomarker analyses. Nevertheless, comprehensive longitudinal data on non-AD dementia progression cofactors remain limited. This study delves into the temporal course and prognosis of VaD, FTD, LBD, and PDD. It scrutinizes the impact of functional, neuropsychiatric, and medical factors on prognosis, explores the correlation between hippocampal atrophy and prognosis, assesses the influence of white matter intensities, and identifies primary covariates influencing slow or rapid progression in non-Alzheimer’s dementias.

## 2. Materials and methods

### 2.1. Data collection and patient selection

Patients for this study were recruited from a dementia database comprising individuals who had sought consultation at the Dementia Outpatient Clinic of the Neurology Department at Mersin University Medical Faculty between 2000 and 2022, under the supervision of the same senior author (AO). The diagnoses of non-Alzheimer’s dementias adhered to specific criteria for VaD, FTD, LBD, and PDD. These diagnoses were supported by clinical evaluations, neuroimaging, and biomarker assessments (3–6, 8).

Upon obtaining written approval from the ethics committee and the relevant institutional permissions, the study commenced (decision no: 2023/28, date: 10.03.2023). All patients included in the study underwent comprehensive evaluations performed by the same clinic, under the supervision of a single author (AO), with regular quarterly visits. These visits played a pivotal role in thorough data collection, thus contributing to a comprehensive grasp of disease progression. Neuropsychiatric assessments occurred at alternate visits, resulting in an average frequency of twice a year, and were diligently recorded in the dedicated database.

In addition to routine neuropsychiatric assessments, each patient engaged in several other visits as part of this study, specifically addressing medical or medication-related concerns. Within this framework, the attending physician not only prescribed necessary medications but also ordered pertinent laboratory tests if deemed necessary. These proactive medical interventions ensured a comprehensive evaluation of each patient’s health status and requirements.

A crucial aspect of patient evaluation encompassed a systematic approach to differential diagnosis. Following an exhaustive neurological examination, patients underwent a series of essential laboratory procedures to facilitate accurate diagnosis. Subsequently, each patient underwent a comprehensive neuropsychological examination employing standardized methodologies. Significantly, the electronic data recording system within the Turkish Alzheimer’s Working Group’s electronic database,^1^ developed under esteemed leadership, played a pivotal role in documenting and organizing acquired data.

This comprehensive neuropsychological evaluation spanned various cognitive domains, including functional capacity, cognition, numerical range, calculation, abstraction, Word Memory Test (WMT), Clock Drawing Test (CDT), and the Global Deterioration Scale (GDS). Methodologies for these tests have been detailed previously (9), ensuring consistency and accuracy in the assessment process.

These meticulous evaluation processes, facilitated by standardized assessments and comprehensive data recording, form the bedrock for the robustness and reliability of our findings. Through maintaining consistent and thorough evaluations, our aim was to capture the nuanced trajectories of disease progression among the non-Alzheimer’s dementia cases under investigation.

To differentiate Non-AD Dementias from other causes of dementia, a neuroimaging protocol involving MRI or CT was employed for differential diagnosis. In certain cases, standardized SPECT/PET investigations were conducted when necessary (to assist in the differential diagnosis of various dementia subtypes, limited to clinical cases meeting our social insurance system rules, such as those with a positive family history or encephalopathy).

This study encompassed clinical dementia cases (GDS 3 or higher) with at least three comprehensive evaluation visits, including eligible MRI scans for radiological assessment, and complete biochemical screenings for validation. We included GDS 3 cases (also referred to as mild cognitive impairment) to assess longitudinal disease courses. Exclusion criteria encompassed the presence of known inflammatory, infectious, or immune diseases causing cognitive disturbances, overlapping syndromes (e.g., AD plus vascular dementia, motor neuron disorders), comorbid neuropsychiatric disorders (e.g., epilepsy, previously diagnosed psychotic disorders, dependency), major head trauma, severe renal or hepatic failure, recent severe hemodynamic disturbances (e.g., decompensated heart failure, shock, acute myocardial ischemia), residing in nursing homes or palliative care units, and refusal by patients or their legal representatives to participate in the study. Moreover, patients residing in nursing homes or receiving palliative care were excluded due to legal restrictions. Due to the nature of neuropsychiatric test evaluation, only samples with formal education were included in the analysis. Furthermore, patients who were bedridden or in the advanced stages of dementia were not brought to the outpatient clinic by their caregivers and hence had to be excluded from the study.

### 2.2. Statistical analysis

The study employed descriptive statistics, one-way ANOVA, chi-square tests, and repeated measurements ANOVA. Patient clusters and their characteristics were identified through group-based trajectory modeling. The analysis utilized the STATA Plugin. Further details are outlined elsewhere (9).

## 3. Results

The study encompassed 196 patients with non-Alzheimer’s disease (non-AD) dementia, divided into categories including VaD, FTD, LBD, and PDD. A thorough examination was conducted on their demographic and clinical features. Memory dysfunction was a common thread across all types, while behavioral issues, sleep problems, and language difficulties displayed variations. Furthermore, comorbid medical conditions were prevalent in all the groups.

**Figure 1:**
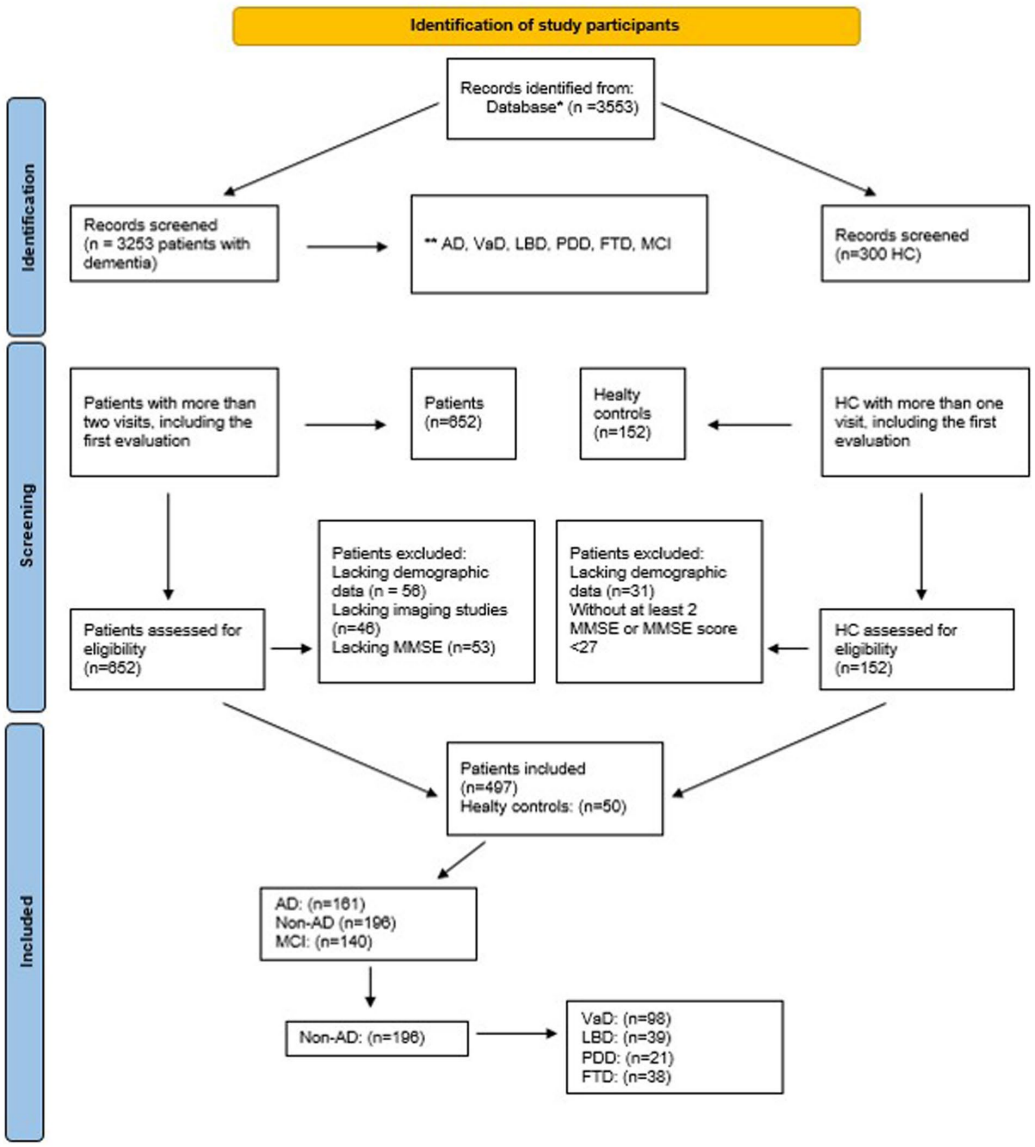
Follow-chart of study sample, *Epikriz.com (Turkish Alzheimer Database, Mersin Branch), *AD, Alzheimer’s disease; VaD, Vascular dementia; LBD, Lewy body dementia; PDD, Parkinson’s disease with dementia; FTD, Frontotemporal dementia; MCI, Mild cognitive impairment; MMSE, mini-mental state examination; HC, Healthy controls.

The trajectory analysis unveiled the presence of two distinct subgroups characterized by slow and fast prognosis. The predictors of disease progression included hippocampal atrophy, BDLAS, EDLAS scores, and alcohol usage during the initial visit. A visual representation of the recruited data and study details can be observed in Figure 1.

### 3.1. Follow-up duration and non-AD dementia types

The average follow-up duration for non-AD cases was 62.57 ± 33.45 months (ranging from 11 to 198 months). Comparatively, healthy controls consisting of 50 individuals had an average follow-up duration of 55.04 ± 41.98 months (ranging from 6 to 163 months). As depicted in Table 1, our data highlights that within the realm of non-AD dementia cases, vascular dementia (VaD) exhibited the highest prevalence (50%), followed by Lewy body disease (LBD, 19.39%) and Parkinson’s disease dementia (PDD, 10.71%).

**Table 1:** Demographic and clinical features of study sample.

	VaD *n* = 98 (50%)	FTLD *n* = 38 (19.39%)	LBD *n* = 39 (19.9%)	PDD *n* = 21 (10.71%)	All Non-AD *n* = 196 (100%)	*p*
Age (mean ± SD)	72.63 ± 8.37	64.31 ± 10.27	72.31 ± 8.05	73.28 ± 6.85	71.02 ± 9.13	<0.0001
Gender						0.03863
Male 97 (49.49%)	52 (53.06%)	11 (28.95%)	23 (58.97%)	11 (52.38%)	97 (49.49%)	
Female 99 (50.51%)	46 (46.94%)	27 (71.05%)	16 (41.03%)	10 (47.62%)	99 (50.51%)	
Main presentation *n* (%)						
Memory dysfunction	36 (36.73%)	19 (50%)	21 (53.85%)	6 (28.57%)	82 (41.83%)	0.11561
Behavioral problem	-	2 (5.26%)	4 (10.26%)	1 (4.76%)	7 (3.57%)	0.01232
Sleep problem	40 (40.82%)	13 (34.21%)	15 (38.46%)	8 (38.09%)	76 (38.77%)	0.91642
Language problem	63 (64.28%)	27 (71.05%)	27 (69.23%)	16 (76.19%)	133 (67.86%)	0.69308
Disorientation	36 (36.73%)	15 (39.47%)	20 (51.28%)	10 (47.62%)	81 (41.33%)	0.41647
Comorbid medical problems *n* (%)						
Hypertension	51 (52.04%)	11 (28.95%)	17 (43.59%)	10 (47.62%)	89 (45.41%)	0.11228
Diabetes Mellitus	23 (23.46%)	3 (7.89%)	6 (15.38%)	6 (28.57%)	38 (19.39%)	0.12232
Coronary Artery Diseases	34 (34.69%)	4 (10.53%)	11 (28.20%)	7 (33.33%)	56 (28.57%)	0.04401
Stroke	9 (9.18%)	1 (2.63%)	-	2 (9.52%)	12 (6.12%)	0.05256
Family history of dementia n (%)	40 (40.81%)	14 (36.84%)	18 (46.15%)	10 (47.62%)	82 (41.84%)	0.79671
Family history of vascular disease	37 (37.75%)	12 (31.58%)	14 (35.90%)	13 (61.90%)	76 (38.77%)	0.12496
Living alone	17 (17.34%)	10 (26.31%)	5 (12.82%)	1 (4.76%)	33 (16.84%)	0.16513
Self-care problem	21 (21.42%)	10 (26.31%)	12 (30.77%)	5 (23.81%)	48 (24.49%)	0.70507
Incontinence	27 (27.55%)	5 (13.16%)	6 (15.38%)	10 (47.62%)	48 (24.49%)	0.01195
Appetite/weight	18 (18.37%)	8 (21.05%)	7 (17.95%)	2 (9.52%)	35 (17.86%)	0.73476
Executive dysfunction	29 (29.59%)	8 (21.05%)	18 (46.15%)	6 (28.57%)	61 (31.12%)	0.10783

VaD, Vascular dementia; FTLD, Frontotemporal Lobar Degeneration; LBD, Lewy body dementia; PDD, Parkinson’s disease dementia.

### 3.2. Demographic differences and clinical presentations

Significant age disparities were observed among the groups, with frontotemporal dementia (FTD) patients having the youngest mean age (64.31), while PDD patients were the oldest (mean = 73.28). Gender variations were also noted; LBD and PDD exhibited a higher percentage of female participants compared to VaD and FTD. In terms of family histories of dementia and comorbid medical conditions, the groups generally displayed similarities, except for an elevated incidence of coronary artery disease (CAD) in VaD and PDD cases.

### 3.3. Symptom prevalence and presentation

Across the spectrum of non-AD dementia types, memory dysfunction was prominent (ranging from 36.73% in VaD to 76.19% in PDD), indicating its early manifestation in various forms of dementia. Behavioral issues, encompassing personality changes, mood swings, and agitation, affected 40.82% in VaD, 34.21% in FTD, 38.09% in LBD, and 38.46% in PDD, significantly impacting individuals’ quality of life. Sleep problems, such as insomnia and altered sleep–wake cycles, were particularly pronounced in FTD and LBD (71.05 and 69.23%, respectively). Notably, FTD also exhibited distinct challenges with language (71.05%). Disorientation was prevalent across different dementia types, ranging from 36.73% in VaD to 51.28% in FTD.

### 3.4. Cognitive and functional assessments

A comprehensive analysis of cognitive and functional assessments highlighted substantial differences between visits. For VaD cases, BDLAS scores reached their peak during the third visit, while EDLAS scores exhibited the highest values during the first visit. Conversely, MMSE scores were at their lowest during the third visit. Among FTD patients, both BDLAS and EDLAS scores displayed significant differences between visits, with the highest mean values recorded during the third and first visits, respectively. MMSE scores also demonstrated significant differences between visits, reaching their lowest mean value during the third visit. In cases of LBD, EDLAS scores exhibited significant differences between visits, and MMSE scores displayed significant variations, indicating the lowest mean value during the third visit. PDD patients demonstrated notable discrepancies in MMSE scores between visits, with the highest mean value observed during the first visit (refer to Tables 2–5).

**Table 2:** Neuropsychiatric evaluation of the cases with VaD.

	First visit	Second visit	Third visit	*p*
Interval of the visits (mean ± SD) (min-max)	26.44 ± 21.19^*^ (3–120)	11.11 ± 11.38 (3–90)	21.38 ± 15.11^**^ (3–60)	**<0.00001**
BDLAS	2.01 ± 1.30 (0–6.5)	2.07 ± 1.41 (0–8)	3.18 ± 1.84 (0–7.5)	**0.009560**
EDLAS	17.46 ± 6.17 (1-23)	16.91 ± 6.38 (0–23)	12.24 ± 6.96 (1-23)	**0.000990**
MMSE	23.33 ± 5.09 (7–30)	22.45 ± 5.90 (4–30)	18.84 ± 7.09 (1–30)	**<0.00001**
Digit forward	4 (2–7)	4 (0–7)	4 (0–7)	0.28136
Digit backward	2 (0–4)	2 (0–6)	2 (0–5)	**0.00080**
Calculation	5 (0–5)	5 (0–5)	3 (0–5)	**0.00049**
Abstraction	3 (0–3)	3 (0–3)	3 (0–3)	**0.00077**
WMT- 1	2 (0–5)	2 (0–7)	2 (0–5)	0.40130
WMT-2	3 (0–7)	3 (0–7)	3 (0–6)	0.21574
WMT-3	4 (0–7)	4 (0–9)	3 (0–10)	0.40111
WMT- recall	0 (0–5)	0 (0–7)	0 (0–7)	0.47478
WMT-recognition	15 (0–20)	14 (0–20)	11 (0–20)	**0.00218**
BNT	13 (0–15)	12 (1–15)	12 (0–15)	**0.000017**
CDT	5 (0–10)	5 (0–10)	4 (0–10)	0.13269
GDS	3 (2–5)	4 (2–6)	5 (2–7)	**<0.00001**
Comprehension	6 (1–10)	6 (0–6)	3 (0–6)	0.06650
Visual memory score	9 (0–11)	7 (0–11)	7 (0–11)	0.46678
Visual memory recall	2 (0–10)	1 (0–9)	0 (0–11)	0.06478

BDLAS, Barthel Daily Living Activities Scale; EDLAS, Elderly Daily Living Activity Scale; MMSE, Minimental State Examination; WMT, Word memory test; BNT, Boston naming test; CDT, Clock Driving Test; GDS, Global Deterioration Scale.

* First visit duration means that duration of the first presenting symptom.

** Total follow up duration in our outpatient clinic is 58.94 ± 30.56, min 11 max 169 months. Bold characters reflect statistically significant values below *p* < 0.05.

**Table 3:** Neuropsychiatric evaluation of the cases with FTLD.

	First visit	Second visit	Third visit	*p*
Interval of the visits (mean ± SD) (min-max)	29.53 ± 26.25^*^ (3–120)	11.66 ± 10.25 (4–60)	23.92 ± 17.02^**^ (3–63)	**0.000704**
BDLAS	1.86 ± 1.23 (0–5)	2.07 ± 1.18 (0.50–4.50)	4.57 ± 2.19 (1.50–8)	**<0.00001**
EDLAS	19 ± 4.68 (4-23)	18.15 ± 5.27 (6–23)	9.54 ± 7.88 (0–22)	**0.000017**
MMSE	21.16 ± 7.40 (4–30)	19.08 ± 8.11 (0–30)	13.24 ± 9.72 (0–30)	**<0.00001**
Digit forward	4 (2–6)	4 (3–6)	4 (0–5)	0.60037
Digit backward	3 (0–4)	2 (0–4)	2 (0–5)	0.80887
Calculation	4 (0–5)	2 (0–5)	2 (0–5)	0.17502
Abstraction	3 (0–3)	3 (0–3)	2 (0–3)	0.07844
WMT-1	3 (0–6)	2 (0–5)	2 (0–5)	0.971964
WMT-2	4 (0–5)	3 (0–10)	3 (0–8)	0.580827
WMT-3	4 (0–7)	4 (0–10)	3 (0–8)	0.494226
WMT-recall	0 (0–6)	0 (0–8)	0 (0–6)	**0.021657**
WMT-recognition	14 (0–19)	13 (0–20)	10 (0–20)	0.130017
BNT	12 (2–15)	11 (7–15)	9 (0–15)	**0.00040**
CDT	4 (0–10)	5 (0–10)	3 (0–10)	0.426309
GDS	3 (3–5)	4 (3–6)	5 (4–7)	**<0.00001**
Comprehension	6 (1–6)	3 (1–6)	1 (0–6)	0.08209
Visual memory score	8 (1–11)	8 (7–11)	7 (0–10)	NA
Visual memory recall	2 (0–11)	5 (1–7)	0 (0–6)	NA

BDLAS, Barthel Daily Living Activities Scale; EDLAS, Elderly Daily Living Activity Scale; MMSE, Minimental State Examination; WMT, Word memory test; BNT, Boston naming test; CDT, Clock Driving Test; GDS, Global Deterioration Scale.

* First visit duration means that duration of the first presenting symptom.

** Total follow up duration in our outpatient clinic is 65.10 ± 29.61, min 31 to max 160 months. Bold characters reflect statistically significant values below *p* < 0.05.

**Table 4:** Neuropsychiatric evaluation of the cases with LBD.

	First visit	Second visit	Third visit	*p*
Interval of the visits mean ± SD (min-max)	32.85 ± 26.97^*^ (3–120)	8.92 ± 6.82 (3–45)	22.05 ± 20.45^**^ (3–91)	**0.000001**
BDLAS	2.83 ± 1.20 (0–7.5)	2.83 ± 2.34 (0–8)	3.62 ± 2.41 (0–8)	0.532782
EDLAS	15.68 ± 7.33 (2-23)	14.12 ± 7.86 (1–23)	11.58 ± 8.92 (0–23)	0.201896
MMSE	21.56 ± 7.37 (4–30)	21.20 ± 7.41 (1–30)	19 ± 8.84 (0–30)	**0.004148**
Digit forward	4 (2–7)	4 (2–6)	4 (0–6)	0.89639
Digit backward	2 (0–6)	2 (0–4)	2 (0–5)	0.79283
Calculation	5 (0–5)	5 (0–5)	4 (0–5)	**0.03438**
Abstraction	3 (0–3)	3 (0–3)	3 (0–3)	0.72747
WMT-1	2 (0–5)	2 (0–5)	1 (0–5)	0.19262
WMT-2	4 (0–6)	3 (0–7)	3 (0–8)	0.83602
WMT-3	4 (0–7)	3 (0–9)	2 (0–9)	0.58597
WMT-recall	0 (0–7)	0 (0–5)	0 (0–7)	0.56690
WMT-recognition	16 (0–20)	14 (0–20)	14 (0–19)	0.71228
BNT	13 (1–15)	13 (0–15)	11 (1–15)	0.768967
CDT	6 (0–10)	4 (0–10)	4 (0–10)	0.47910
GDS	4 (2–5)	4 (3–6)	5 (3–7)	**<0.00001**
Comprehension	6 (0–6)	6 (1–6)	6 (0–6)	0.76130
Visual memory score	4 (0–11)	6 (0–11)	5 (0–11)	0.36788
Visual memory recall	2 (0–11)	1 (0–11)	0 (0–8)	0.36788

BDLAS, Barthel Daily Living Activities Scale; EDLAS, Elderly Daily Living Activity Scale; MMSE, Minimental State Examination; WMT, Word memory test; BNT, Boston naming test; CDT, Clock Driving Test; GDS, Global Deterioration Scale.

* First visit duration means that duration of the first presenting symptom.

** Total follow up duration in our outpatient clinic is 63.82 ± 40.12, min 17 to max 198 months. Bold characters reflect statistically significant values below *p* < 0.05.

**Table 5:** Neuropsychiatric evaluation of the cases with PDD.

	First visit	Second visit	Third visit	*p*
Interval of the visits (mean ± SD)	32.14 ± 34.96^*^ (3–120)	10.52 ± 5.97 (3–27)	29.95 ± 24.95^**^ (4–94)	**0.019921**
BDLAS	2.05 ± 1.30 (0.50–5)	2.72 ± 2.23 (0.50–7.50)	2.69 ± 2.52 (0–7)	0.505876
EDLAS	17.33 ± 5.69 (7-23)	13.87 ± 8.40 (0–23)	14.71 ± 9.70 (1-23)	0.165435
MMSE	23.67 ± 4.85 (13–30)	22.81 ± 7.96 (7–30)	19.14 ± 9.35 (0–29)	0.003333
Digit forward	4 (3–6)	4 (0–5)	4 (0–6)	0.54881
Digit backward	3 (0–4)	2 (0–3)	2 (0–5)	0.55382
Calculation	5 (1–5)	5 (0–5)	3 (0–5)	**0.00674**
Abstraction	3 (0–3)	3 (0–3)	3 (1–3)	0.09902
WMT-1	3 (0–5)	3 (0–6)	3 (0–6)	0.58665
WMT-2	4 (0–9)	4 (0–9)	4 (2–7)	0.73380
WMT-3	4 (0–9)	5 (0–9)	4 (0–7)	0.07148
WMT-recall	2 (0–9)	0 (0–6)	1 (0–6)	0.51935
WMT-recognition	15 (6–20)	15 (0–20)	18 (0–20)	0.83570
BNT	14 (2–15)	14 (0–15)	12 (1–15)	0.113404
CDT	4 (0–10)	6 (0–10)	5 (0–10)	0.11390
GDS	3 (3–4)	4 (3–5)	5 (3–6)	**<0.00001**
Comprehension	6 (5–6)	6 (1–6)	6 (1–6)	0,36,788
Visual memory score	10 (5–11)	5 (2–7)	5 (0–11)	NA
Visual memory recall	9 (3–11)	3 (0–7)	2 (0–11)	NA

BDLAS, Barthel Daily Living Activities Scale; EDLAS, Elderly Daily Living Activity Scale; MMSE, Minimental State Examination; WMT, Word memory test; BNT, Boston naming test; CDT, Clock Driving Test; GDS, Global Deterioration Scale.

* First visit duration means that duration of the first presenting symptom.

** Total follow up duration in our outpatient clinic is 72.62 ± 38.94, min 11 to max 159 months. Bold characters reflect statistically significant values below *p* < 0.05.

### 3.5. Disease progression predictors

Hippocampal atrophy emerged as a significant predictor of disease progression in non-AD dementias, as highlighted in Table 6. However, other factors, including epilepsy, extrapyramidal symptoms, thyroid dysfunction, comorbidities, and alcohol usage, did not significantly predict progression. Remarkably, regular alcohol consumption was identified as a substantial predictor of disease progression.

**Table 6:** Laboratory evaluation of the cases with Non-Alzheimer Dementia.

	VaD *n* = 98 (50%)	FTLD *n* = 38 (19.39%)	LCD/PDD *n* = 60 (30.61%)	Total *n* = 196 (100%)
APOE genotype *n*(%)				
E3/E4	1 (25%)	1 (20%)	1 (16.67%)	3 (20%)
E3/E3	3 (75%)	1 (20%)	4 (66.67%)	8 (53.33%)
E2/E3	0 (0%)	3 (60%)	1 (16.67%)	4 (26.67)
Hippocampal atrophy				
Grade 1	57 (58.16%)	15 (39.47%)	18 (30%)	90 (45.92%)
Grade 2	33 (33.67%)	19 (50%)	24 (40%)	76(38.77%)
Grade 3	8 (8.16%)	4 (10.53%)	18 (30%)	30 (15.31%)
Fazekas grading				
Grade 0	1 (1.03%)	17 (44.74%)	17 (28.33%)	35 (17.95%)
Grade 1	16 (16.49%)	20 (52.63%)	26 (43.33%)	62 (31.79%)
Grade 2	46 (47.42%)	1 (2.63%)	13 (21.67%)	60 (30.77%)
Grade 3	34 (35.05%)	0 (0%)	4 (6.67%)	38 (19.49%)
Associated				
Hydrocephalus	4 (4.08%)	1 (2.63%)	2 (3.33%)	7 (3.61%)
Epilepsy	23 (23.47%)	9 (23.68%)	17 (28.33%)	49 (25%)
Extrapyramidal symptoms	10 (10.20%)	4 (10.53%)	50 (83.33%)	64 (32.65%)

### 3.6. Group-based trajectory analysis

**Figure 2:**
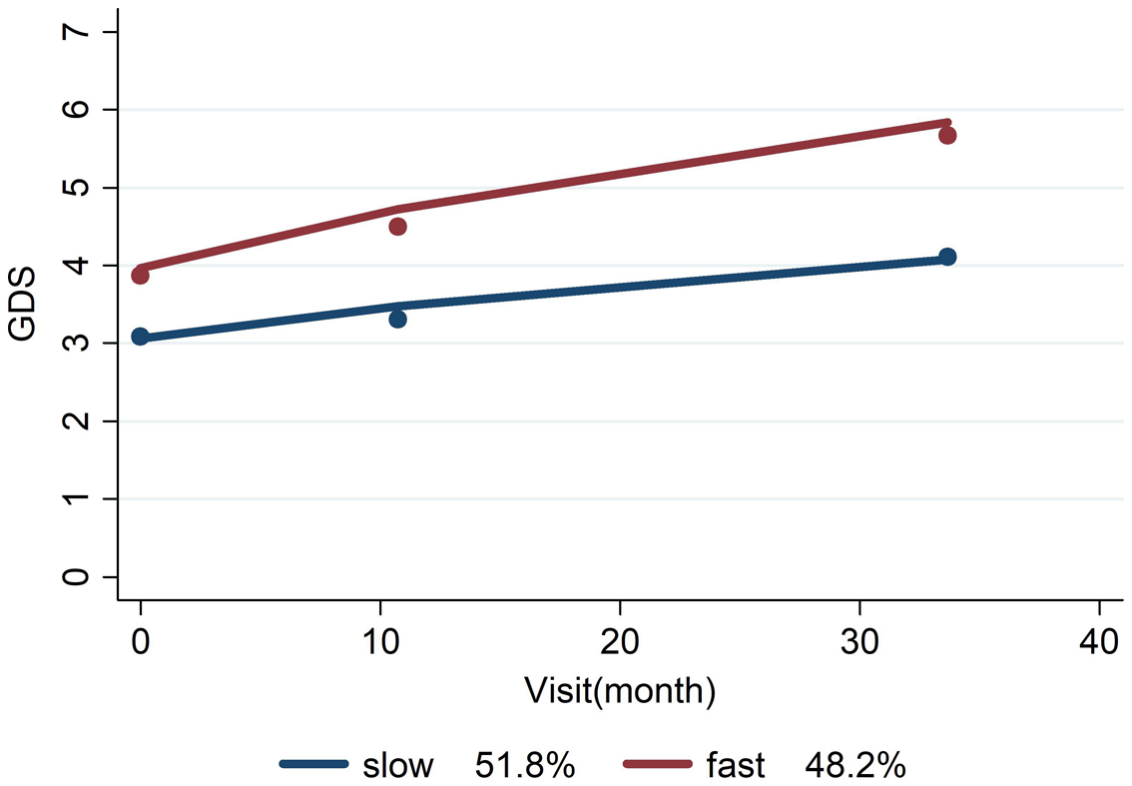
Slow/fast prognosis of non-AD dementias.

Detailed in Table 7 and Figure 2, the group-based trajectory analysis partitioned non-AD cases into two subgroups, denoted as Slow and Fast progression, based on the rate of disease advancement. This analysis underscored that hippocampal atrophy significantly predicted disease progression. Moreover, regular alcohol usage emerged as a predictive factor, whereas current alcohol usage did not significantly predict progression.

**Table 7:** Observed variables on the prognosis of Non-AD dementias.

	Slow (*n* = 100, 51%)	Fast (*n* = 96, 49%)	Trajectory model		
Fazekas grading	n	n	Coeff.	Std. Error	p
0	13	22	−0.10940	0.15801	0.4890
1	32	30			
2	36	24			
3	18	20			
Hippocampal atrophy			0.79410	0.24064	**0.0010**
1	58	32			
2	34	42			
3	8	22			
BDLAS-first visit	1.640 ± 1.170 (0–5)	2.704 ± 1.589 (0–7.5)	0.65850	0.15916	**<0.0001**
BDLAS-second visit	1.746 ± 1.262 (0–7)	2.888 ± 1.951 (0–8)			
BDLAS-Third visit	2.534 ± 1.789 (0–7)	4.254 ± 2.110 (0–8)			
EDLAS-First visit	19.261 ± 4.979 (2–23)	15.365 ± 6.697 (1–23)	−0.14398	0.03618	**0.0001**
EDLAS-Second visit	18.567 ± 5.702 (0–23)	13.557 ± 7.200 (0–23)			
EDLAS-Third visit	15.418 ± 7.081 (1–23)	8.84 ± 7.141 (0–23)			
Epilepsy			0.18991	0.37882	0.6163
No	75	72			
Yes	25	24			
Extrapyramidal symptoms			0.28132	0.35489	0.4283
No	72	60			
Yes	28	36			
Thyroid dysfunction			−0.45160	0.55121	0.4130
No	87	86			
Yes	13	10			
Coronary artery disease			0.15207	0.39435	0.6999
No	71	69			
Yes	29	27			
Diabetes mellitus			−0.09749	0.43671	0.8234
No	80	78			
Yes	20	18			
Hypertension			0.08844	0.35742	0.8047
No	57	50			
Yes	43	46			
Stroke			0.11816	0.69637	0.8653
No	94	90			
Yes	6	6			
Hyperlipidemia			−0.72090	0.45881	0.1167
No	77	80			
Yes	23	16			
Sleep disorders			0.56954	0.34356	0.0979
No	67	53			
Yes	33	43			
Smoking			0.23667	0.31038	0.4461
Current smoker	8	7			
Non smoker	71	67			
Ex-smoker	21	22			
Regular alcohol usage			1.16135	0.52985	**0.0288**
Current-	12	2			
Non-	84	88			
Ex-	4	6			
Family history of dementia					
No	60	54	−0.06610	0.31666	0.8347
Yes	40	42			

Bold characters reflect statistically significant values below *p* < 0.05.

### 3.7. Modeling potential stakeholders

The Group-Based Trajectory Analysis successfully delineated a dependable segregation of non-AD cases into subgroups characterized by Slow and Fast prognoses, visually represented in Figure 2. This subdivision holds paramount importance in comprehending the diverse rates of disease progression.

## 4. Discussion

To comprehensively investigate the clinical and neuroimaging aspects of patients with non-Alzheimer’s dementias, including vascular dementia (VaD), frontotemporal dementia (FTD), Lewy body disease with cortical predominant (LCD), and Parkinson’s disease dementia (PDD), we gathered data from a sizable patient sample. Our analysis delved into their demographic attributes, cognitive performance, and imaging outcomes. Additionally, we explored the trajectories of disease progression within each group, identifying potential factors influencing the rate of decline. The findings from this study furnish an inclusive panorama of the clinical and imaging attributes associated with non-Alzheimer’s dementias, carrying significant implications for their diagnosis and management.

The results from our investigation align with prior literature that has observed disparities in demographic characteristics across various subtypes of non-Alzheimer’s dementia. For example, VaD has been noted to be more prevalent in elderly adults, especially women (7). Conversely, LBD has been linked to a higher occurrence in women in some studies (10), while others report no gender-related differences (11). Moreover, FTD is recognized for affecting individuals at a younger age compared to other dementia types (12). These findings underscore the role of demographic variables in the identification and care of non-Alzheimer’s dementias.

Our study outcomes indicate that VaD ranked as the most prevalent dementia type, followed by LBD and PDD. This outcome is consistent with previous research indicating that VaD constitutes the most common dementia after Alzheimer’s disease (AD), accounting for approximately 20–30% of all dementia cases. In contrast, LBD and PDD are less frequent yet substantial contributors to dementia cases (13, 14). Furthermore, our study unveiled significant distinctions in age and gender among the groups, with FTD patients being the youngest, and LBD and PDD displaying a higher proportion of female participants compared to VaD and FTD. These observations corroborate previous studies highlighting the significance of age and gender in the manifestation and diagnosis of diverse dementia types (15, 16). We also ascertained that family histories of dementia and comorbid medical issues did not differ significantly between the groups, except for a notably elevated occurrence of coronary artery disease (CAD) in VaD and PDD cases compared to others. This concurs with earlier research indicating a link between dementia and various medical conditions like hypertension, diabetes, and cardiovascular ailments (17, 18).

Behavioral issues constituted the predominant initial presentation across all groups except for PDD, where language difficulties took precedence. This correspondence with earlier studies underscores the commonality of behavioral symptoms in different dementia types, particularly in VaD and LBD (19). Parameters such as living alone, self-care concerns, and executive dysfunction were uniform across the groups. However, incontinence was significantly more prevalent in PDD in comparison to other groups. This concurs with existing literature suggesting that executive dysfunction and incontinence are typical features, especially in PDD (20, 21).

Vascular dementia, characterized by cognitive and neuropsychiatric symptoms linked to cerebrovascular disease, is a prevalent cause of dementia (13). Our study demonstrates that BDLAS scores, EDLAS scores, MMSE scores, Digit backward scores, Calculation scores, Word Memory Test (WMT-2), WMT-recall, WMT-recognition scores, Boston Naming Test (BNT) scores, and Global Deterioration Scale (GDS) scores displayed significant variance between follow-up visits. In contrast, Digit forward scores, Abstraction scores, Clock Drawing Test (CDT) scores, Comprehension scores, Visual Memory Score, and Visual Memory Recall scores did not exhibit significant differences. This coherence with prior research underscores that VaD is marked by an array of neuropsychiatric symptoms encompassing cognitive impairment, executive dysfunction, and behavioral and psychological manifestations (22). Specifically, our findings suggest that VaD patients manifest noteworthy fluctuations in their cognitive and functional capacities, as measured by diverse tests employed in our study. BDLAS and EDLAS scores, gauging functional capacity and daily living activities, respectively, experienced significant variance between visits, with the highest mean value during the third visit. This conforms with earlier investigations indicating that functional impairment is a characteristic aspect of VaD, tending to deteriorate with time (23). Similarly, the MMSE scores, a widely employed cognitive impairment screening tool, displayed significant variations between visits, with the lowest mean value during the third visit. This aligns with previous studies highlighting progressive cognitive impairment as a salient feature of VaD (24). Furthermore, parameters like Digit backward scores, Calculation scores, WMT-2, WMT-recall, WMT-recognition scores, and BNT scores, evaluating different facets of executive function, exhibited noteworthy differences between visits, with the first or third visit demonstrating the highest mean value. This parallels with existing evidence that underscores executive dysfunction as a hallmark of VaD (25).

Our findings highlight that Fazekas grading and hippocampal atrophy significantly foretell disease progression in non-AD dementias. This resonance with prior research underlines the association between neuroimaging markers, such as white matter hyperintensities (Fazekas grading) and hippocampal atrophy, and disease severity and progression in various non-AD dementias (8, 26).

However, factors like epilepsy, extrapyramidal symptoms, thyroid dysfunction, coronary artery disease, diabetes mellitus, hypertension, stroke, hyperlipidemia, sleep disorders, smoking, and family history of dementia did not emerge as significant predictors of disease progression in our study. It is important to note that the lack of statistical significance does not necessarily negate potential associations, as sample size or study design could influence results. In-depth investigations with larger sample sizes and longitudinal methodologies are warranted to explore these factors’ potential roles in non-AD dementias.

Furthermore, our study delved into the relationship between BDLAS and EDLAS scores during different visits and disease progression. The BDLAS scores during the first and second visits exhibited significant associations with disease progression, whereas the BDLAS score during the third visit did not show a significant correlation. Similarly, the EDLAS scores during the first and second visits were significantly linked to disease progression, whereas the EDLAS score during the third visit did not attain significance. These findings indicate that assessments of functional disability, as gauged by BDLAS and EDLAS, offer valuable insights into early-stage disease progression in non-AD dementias (27).

Regarding alcohol usage, regular consumption emerged as a significant predictor of disease progression, while current alcohol usage did not exhibit a significant connection. The intricate relationship between alcohol consumption and dementia progression necessitates further exploration. Certain studies posit a protective effect of moderate alcohol consumption on cognitive decline, while heavy alcohol usage is associated with an elevated risk of dementia (28). Unraveling the specific mechanisms underpinning these associations and their implications for non-AD dementias warrants future investigation.

### 4.1. Implications

The primary implications of our study encompass:

- Offering an extensive overview of the clinical and imaging attributes of non-Alzheimer’s dementias, encompassing VaD, FTD, LBD, and PDD.
- Presenting some of the longest-reported follow-up durations (up to 198 months) for these cases in the literature.
- Underscoring the value of tailored neuropsychiatric assessment, including functional capacity evaluation, in diagnosing and managing diverse non-AD dementia subtypes. Functional disability assessments, represented by BDLAS and EDLAS scores, provide critical insights into early-stage disease progression in non-AD dementias.
- Highlighting specific comorbidities, like CAD and epilepsy, associated with distinct non-AD dementia subtypes, thereby influencing diagnosis and management.
- Illuminating the prevalence of behavioral issues across non-AD dementias, particularly in VaD and LBD, with language problems more prominent in PDD.
- Identifying neuroimaging markers, such as Fazekas grading and hippocampal atrophy, as significant predictors of disease progression in non-AD dementias, reinforcing their role in assessing disease severity.
- Suggesting that regular alcohol consumption might significantly predict disease progression, thereby emphasizing the need for further exploration of the intricate link between alcohol use and dementia.

### 4.2. Limitations

Despite its contributions, our study bears the following limitations:

- Sample Size: Despite the inclusion of a substantial patient sample, relatively smaller subgroup sizes, such as FTD, could limit the generalizability of our findings.
- Retrospective Design: The retrospective nature of our study based on existing records may introduce biases and limitations in data collection, impacting accuracy and completeness.
- Selection Bias: Inclusion criteria and recruitment methods might skew the sample, potentially excluding certain individuals or favoring specific subgroups, thus limiting generalizability.
- Missing Data: Longitudinal assessments over an extended period may entail missing data, influencing analysis and interpretation accuracy.
- Assessment Tools: Dependency on specific assessment tools introduces limitations in terms of sensitivity, specificity, and standardization.
- Unaccounted Factors: Unmeasured or unknown factors influencing non-AD dementia progression may exist, potentially impacting associations.
- Single-Center Study: Conducting the study at a single center may limit generalizability to diverse healthcare settings and populations.

### 4.3. Conclusion

In summary, our study of non-Alzheimer’s dementias, encompassing VaD, FTD, LBD, and PDD, offers valuable insights into prognostic factors. It underscores the significance of demographic attributes, cognitive performance, and imaging findings in diagnosing and managing these conditions. VaD emerged as the most prevalent dementia type, followed by LBD and PDD. Age and gender variations underscored the relevance of these factors in comprehending dementia presentation and progression. Moreover, our study highlights the predictive role of Fazekas grading and hippocampal atrophy in disease progression for non-AD dementias. In sum, this research advances our understanding of non-Alzheimer’s dementias and pinpoints avenues for further research and clinical attention in their diagnosis and management.

## Data availability statement

The datasets presented in this article are not readily available because of ethical and privacy restrictions. Requests to access the datasets should be directed to the corresponding author/s.

## Ethics statement

The studies involving humans were approved by Toros University Ethical Committy - decision no: 2023/28, date: 10.03.2023. The studies were conducted in accordance with the local legislation and institutional requirements. Written informed consent for participation was not required from the participants or the participants’ legal guardians/next of kin in accordance with the national legislation and institutional requirements.

## Author contributions

RG: data collection, conception, design, and revision of the article. RG, AÖ, and NÖ: data collection. RG, AÖ, NÖ, and BT: analysis and drafting of the article. RG: revision of the article and approved the final version. All authors contributed to the article and approved the submitted version.

## Footnotes

^1^ www.epikriz.com/dementiadataset

## References

[1] Guerreiro R Gibbons E Tábuas-Pereira M Kun-Rodrigues C Santo GC Bras J. Genetic architecture of common non-Alzheimer’s disease dementias. Neurobiol Dis. (2020) 142:104946. doi: 10.1016/j.nbd.2020.104946, PMID: 32439597 PMC8207829

[2] Smith EE Biessels GJ De Guio F de Leeuw FE Duchesne S Düring M. Harmonizing brain magnetic resonance imaging methods for vascular contributions to neurodegeneration. Alzheimers Dement. (2019) 11:191–204. doi: 10.1016/j.dadm.2019.01.002, PMID: 30859119 PMC6396326

[3] Sachdev P Kalaria R O’Brien J Skoog I Alladi S Black SE. Internationlal Society for Vascular Behavioral and Cognitive Disorders. Diagnostic criteria for vascular cognitive disorders: a VASCOG statement. Alzheimer Dis Assoc Disord. (2014) 28:206–18. doi: 10.1097/WAD.0000000000000034, PMID: 24632990 PMC4139434

[4] Alafuzoff I Pikkarainen M Neumann M Arzberger T Al-Sarraj S Bodi I. Neuropathological assessments of the pathology in frontotemporal lobar degeneration with TDP43-positive inclusions: an inter-laboratory study by the BrainNet Europe consortium. J Neural Transm (Vienna). (2015) 122:957–72. doi: 10.1007/s00702-014-1304-1, PMID: 25239189

[5] McKeith IG Boeve BF Dickson DW Halliday G Taylor JP Weintraub D. Diagnosis and management of dementia with Lewy bodies: fourth consensus report of the DLB consortium. Neurology. (2017) 89:88–100. doi: 10.1212/WNL.0000000000004058, PMID: 28592453 PMC5496518

[6] Uysal-Cantürk P Hanağası HA Bilgiç B Gürvit H Emre M. An assessment of Movement Disorder Society task force diagnostic criteria for mild cognitive impairment in Parkinson’s disease. Eur J Neurol. (2018) 25:148–53. doi: 10.1111/ene.13467, PMID: 28941002

[7] Wolters FJ Ikram MA. Epidemiology of vascular dementia. Arterioscler Thromb Vasc Biol. (2019) 39:1542–9. doi: 10.1161/ATVBAHA.119.311908 31294622

[8] Chang Wong E Chang Chui H. Vascular cognitive impairment and dementia. Continuum. (2022) 28:750–80. doi: 10.1212/CON.0000000000001124, PMID: 35678401 PMC9833847

[9] Özge A Ghouri R Öksüz N Taşdelen B. (2023). Predictive factors for alzheimer’s disease progression: A comprehensive retrospective analysis of 3553 cases with 211 months follow-up. Front Neurol. 14:1239995. doi: 10.3389/fneur.2023.1239995 37693748 PMC10484751

[10] Molano J Boeve B Ferman T Smith G Parisi J Dickson D. Mild cognitive impairment associated with limbic and neocortical Lewy body disease: a clinicopathological study. Brain. (2010) 133:540–56. doi: 10.1093/brain/awp280, PMID: 19889717 PMC2822633

[11] Beach TG Monsell SE Phillips LE Kukull W. Accuracy of the clinical diagnosis of Alzheimer disease at National Institute on Aging Alzheimer disease centers, 2005-2010. J Neuropathol Exp Neurol. (2012) 71:266–73. doi: 10.1097/NEN.0b013e31824b211b, PMID: 22437338 PMC3331862

[12] Huey ED Putnam KT Grafman J. A systematic review of neurotransmitter deficits and treatments in frontotemporal dementia. Neurology. (2006) 66:17–22. doi: 10.1212/01.wnl.0000191304.55196.4d, PMID: 16401839 PMC4499854

[13] O’Brien JT Thomas A. Vascular dementia. Lancet. (2015) 386:1698–706. doi: 10.1016/S0140-6736(15)00463-8 26595643

[14] McKeith IG. Diagnosis and management of dementia with Lewy bodies: Fourth consensus report of the DLB Consortium. Neurol. (2018) 90:300–1. doi: 10.1212/WNL.00000000000049 29438029

[15] Savva GM Wharton SB Ince PG Forster G Matthews FE Brayne C. Age, neuropathology, and dementia. N Engl J Med. (2009) 360:2302–9. doi: 10.1056/NEJMoa0806142 19474427

[16] Riedel BC Thompson PM Brinton RD. Age, APOE and sex: triad of risk of Alzheimer’s disease. J Steroid Biochem Mol Biol. (2016) 160:134–47. doi: 10.1016/j.jsbmb.2016.03.012 26969397 PMC4905558

[17] Iadecola C Yaffe K Biller J Bratzke LC Faraci FM Gorelick PB. Impact of hypertension on cognitive function: a scientific statement from the American Heart Association. Hypertension. (2016) 68:e67–94. doi: 10.1161/HYP.0000000000000053, PMID: 27977393 PMC5361411

[18] Iadecola C Yaffe K Biller J Bratzke LC Faraci FM Gorelick PB. Association between hypertension and dementia: a systematic review and meta-analysis. J Neurol Sci. (2019) 398:50–3. doi: 10.1016/j.jns.2019.01.026 30682521

[19] Aarsland D Ballard C Larsen JP McKeith I. A comparative study of psychiatric symptoms in dementia with Lewy bodies and Parkinson’s disease with and without dementia. Int J Geriatr Psychiatry. (2001) 16:528–36. doi: 10.1002/gps.379 11376470

[20] Hely MA Morris JG Reid WG Trafficante R. Sydney multicenter study of Parkinson’s disease: non-L-dopa-responsive problems dominate at 15 years. Mov Disord. (2005) 20:190–9. doi: 10.1002/mds.20324, PMID: 15551331

[21] Palmer K Di Iulio F Varsi AE Gianni W Sancesario G Caltagirone C. Neuropsychiatric predictors of progression from amnestic-mild cognitive impairment to Alzheimer’s disease: the role of depression and apathy. J Alzheimers Dis. (2010) 20:175–83. doi: 10.3233/JAD-2010-1355, PMID: 20164594

[22] Román GC. Vascular dementia revisited: Diagnosis, pathogenesis, treatment, and prevention. Med Clin North Am. (2002) 86:477–99. doi: 10.1016/s0025-7125(02)00008-1 12168556

[23] Wang J Yu JT Wang HF Meng XF Wang C Tan CC. Pharmacological treatment of vascular dementia: a network meta-analysis of randomized controlled trials. CNS Drugs. (2015) 29:847–63. doi: 10.1007/s40263-015-0281-8, PMID: 26400189 PMC4636994

[24] O’Brien JT Erkinjuntti T Reisberg B Roman G Sawada T Pantoni L. Vascular cognitive impairment. Lancet Neurol. (2003) 2:89–98. doi: 10.1016/s1474-4422(03)00305-3 12849265

[25] Sachdev PS Brodaty H Valenzuela MJ Lorentz L Looi JC Berman K. Clinical determinants of dementia and mild cognitive impairment following ischaemic stroke: the Sydney stroke study. Dement Geriatr Cogn Disord. (2006) 21:275–83. doi: 10.1159/000091047, PMID: 16484805

[26] Veitch DP Weiner MW Aisen PS Beckett LA DeCarli C Green RC. Alzheimer’s disease neuroimaging initiative. Using the Alzheimer’s disease neuroimaging initiative to improve early detection, diagnosis, and treatment of Alzheimer’s disease. Alzheimers Dement. (2022) 18:824–57. doi: 10.1002/alz.12422, PMID: 34581485 PMC9158456

[27] Burton RL O’Connell ME Morgan DG. Cognitive and neuropsychiatric correlates of functional impairment across the continuum of no cognitive impairment to dementia. Arch Clin Neuropsychol. (2018) 33:795–807. doi: 10.1093/arclin/acx112, PMID: 29190312

[28] Peters R Peters J Warner J Beckett N Bulpitt C. Alcohol, dementia and cognitive decline in the elderly: a systematic review. Age Ageing. (2008) 37:505–12. doi: 10.1093/ageing/afn095 18487267

